# Genetic diversity and evolutionary patterns of *Taraxacum kok‐saghyz* Rodin

**DOI:** 10.1002/ece3.7622

**Published:** 2021-05-07

**Authors:** Yan Zhang, Hailong Ren, Xuechao Zhang, Li Wang, Qiang Gao, Abudukeyoumu Abudurezike, Qingqing Yan, Zifeng Lu, Yonggang Wang, Qiuhai Nie, Lin Xu, Zhibin Zhang

**Affiliations:** ^1^ Institute of Crop Germplasm Resources Xinjiang Academy of Agricultural Sciences Urumqi China; ^2^ Guangzhou Academy of Agricultural Sciences Guangzhou China; ^3^ Institute of Agricultural Sciences of the Yili Prefecture Yining China; ^4^ Sanya Crop Breeding Test Center Xinjiang Academy of Agricultural Sciences Sanya China; ^5^ Linglong Beijing Dandelion Technology& Development Co., Ltd. Beijing China; ^6^ State Key Laboratory of Cotton Biology Institute of Cotton Research Chinese Academy of Agricultural Sciences Anyang China

**Keywords:** adaptation, population genetic diversity, population structure, rubber dandelion, SNP

## Abstract

*Taraxacum kok‐saghyz* Rodin (TKS) is an important potential alternative source of natural inulin and rubber production, which has great significance for the production of industrial products. In this study, we sequenced 58 wild TKS individuals collected from four different geography regions worldwide to elucidate the population structure, genetic diversity, and the patterns of evolution. Also, the first flowering time, crown diameter, morphological characteristics of leaf, and scape of all TKS individuals were measured and evaluated statistically. Phylogenetic analysis based on SNPs and cluster analysis based on agronomic traits showed that all 58 TKS individuals could be roughly divided into three distinct groups: (a) Zhaosu County in Xinjiang (population AB, including a few individuals from population C and D); (b) Tekes County in Xinjiang (population C); and (c) Tuzkol lake in Kazakhstan (population D). Population D exhibited a closer genetic relationship with population C compared with population AB. Genetic diversity analysis further revealed that population expansion from C and D to AB occurred, as well as gene flow between them. Additionally, some natural selection regions were identified in AB population. Function annotation of candidate genes identified in these regions revealed that they mainly participated in biological regulation processes, such as transporter activity, structural molecule activity, and molecular function regulator. We speculated that the genes identified in selective sweep regions may contribute to TKS adaptation to the Yili River Valley of Xinjiang. In general, this study provides new insights in clarifying population structure and genetic diversity analysis of TKS using SNP molecular markers and agronomic traits.

## Graphical abstract

In the current study, we have discovered that 58 Taraxacum kok‐saghyz Rodin materials collected from different regions can be classified into three different groups based on SNP markers along with their geographical origins: (a) Zhaosu County in Xinjiang (ZS); (b) Tekes County in Xinjiang (TS); and (c) Tuzkol lake in Kazakhstan. We found that TKS population expansion from ZS to Kazakhstan and TS occurred, as well as gene flow between them. These findings presumed that TKS adaptation to the Yili River Valley of Xinjiang, China.

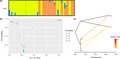

## 1 INTRODUCTION

Natural rubber (NR) is an important high‐performance material, which has incomparable advantages over petroleum‐derived synthetic rubbers in many applications requiring abrasion, heat dispersion, resilience, and other desirable properties (van Beilen & Poirier, 2007). As a valuable biopolymer, NR can be used to manufacture many rubber products, including latex gloves and tires (Cherian et al., 2019). To date, the production of NR in the world mainly comes from the Brazilian rubber tree *Hevea brasiliensis* (Cornish, 2017), and NR is also faced with fluctuating prices and increasing demand with the rapid economic development in many countries (van Beilen & Poirier, 2007). Therefore, we need to find some renewable NR materials to replace petroleum‐derived products. Russian dandelion *Taraxacum kok‐saghyz* Rodin (TKS) is considered to be a promising renewable NR material to replace petroleum‐derived products (Clement‐Demange et al., 2007). It is native to Xinjiang areas in China and Kazakhstan, and often grows in salinized meadows, flood plain meadows, and farmland canals (Krotkov, 1945; Whaley & Bowen, 1947). After recognizing the importance and urgency of developing alternative sources for NR, many countries have collected a large number of TKS germplasm resources and strengthened the basic research on TKS (Luo et al., 2017). For example, Lin et al. (2018) had sequenced the reference genome of TKS. Inulin content, rubber content, and root biomass of TKS population were also evaluated based on agronomic characteristics and molecular markers (Arias et al., 2016). Moreover, genetic resources play an important role in germplasm identification, breeding strategy and crop improvement. Previous studies about the population diversity of TKS have been conducted using eSSRs and gSSRs as markers, such as McAssey et al. (2016) running rudimentary species diversity study of TKS using eSSRs and Nowicki et al. (2019) providing many additional insights using gSSRs on the USDA germplasm of TKS. The evaluation of the genetic diversity and evolutionary patterns in TKS is extremely important for the constitution of repository fields, obtained from the selection of genotypes collected from wild plants; the genetic diversity needs to be assessed and eventually integrated; finally, a rational conservation program for TKS germplasm resources needs to be associated with a genetic diversity evaluation.

High‐throughput molecular markers, such as amplified fragment length polymorphism and random amplified polymorphic DNA, are effective in elucidating and identifying genetic backgrounds (Abdollahi et al., 2015; Bhagyawant, 2016; Cheng et al., 2015; Fu et al., 2015). These marker systems are economical, simple, and automated compared with whole‐genome resequencing, but not numerous enough to saturate large populations. With the rapid development of deep sequencing technologies, the aforementioned shortcomings of these traditional markers are being resolved. These emerging sequencing technologies make it possible for high‐throughput identification of SNPs in many species, including those without reference genomes (Rimbert et al., 2018; Zhou et al., 2017). For example, SNPs, an emerging molecular marker type, are efficient and powerful for population genetics studies. These approaches have facilitated the whole‐genome resequencing for several hundred lines, marker genotyping platforms, the development of high‐density genetic maps, and markers related to agronomic traits (Varshney et al., 2019). Nowadays, we can identify causal genetic features for breeders to perform biological interventions by integrating genotype and phenotype data effectively (Ramstein et al., 2018; Wallace et al., 2018; Zhang et al., 2018), such as genome‐wide association study (GWAS). Moreover, high‐throughput SNP molecular markers have not been used to evaluate the genetic diversity and evolutionary patterns of TKS germplasm resources worldwide so far (Cherian et al., 2019).

In this study, a population evolutionary analysis of 58 TKS individuals collected from different distribution regions was implemented to (a) characterize TKS population genetic structure and (b) identify candidate genes under natural selection for TKS germplasm adaptation to the Yili River Valley based on SNP markers developed using Illumina GBS approach.

## 2 MATERIALS AND METHODS

### 2.1 Germplasm collection and DNA extraction

Fifty‐eight wild TKS individuals were collected from four different geographical regions worldwide (Table S1) (80.77°E, 42.74°N; 81.05°E, 42.76°N; 81.91°E, 43.22°N; 79.97°E, 43.01°N) for Illumina Genotyping‐by‐Sequencing (GBS sequencing). Among them, 15 come from meadow in Zhaosu County of Xinjiang (named population A), 15 come from Tekes River wetland in Zhaosu County of Xinjiang (named population B), 14 come from Tekes River Wetland in Tekes County of Xinjiang (named population C) and 14 come from Tuzkol lake in Kazakhstan (named population D). The distances between populations were at least 4 km, and individuals within one population were sampled in at least 50 m apart. All 58 living individuals were taken from their origins to the experimental field of Yili Prefecture Agricultural Science Research Institute for ball planting in 2017. After 1 year, eight different agronomic traits including leaf thickness (LT), crown diameter (CD), beginning of flowering (BF), leaf length (LL), scape number (SN), leaf width (LW), scape length (SL), and scape diameter (SD) of 58 TKS individuals were investigated with three technical replications. Then, descriptive statistical analysis and cluster analysis of agronomical data were carried out using R package pastecs (https://github.com/phgrosjean/pastecs/issues) and hclust with method=“ward.D2” (https://github.com/mljs/hclust), respectively. The fresh young, healthy leaves of 58 individuals were collected and snapped frozen in liquid nitrogen and then stored at −80°C for GBS sequencing. The genomic DNA was extracted using CTAB methods (Abdel‐Latif & Osman, 2017).

### 2.2 GBS sequencing

The extracted DNA was quantified by a Nanodrop 2000 UV–Vis spectrophotometer (Thermo Fisher Scientific) and then incubated with MseI (New England Biolabs), T4 DNA ligase (NEB), ATP, and the Y‐adapter N containing a barcode. The digestion was conducted at 37°C and heated at 65°C to inactivate the enzymes. Restriction digestion–ligation reactions were completed in the same tube and then further digested with NlaIII (NEB) and EcoRI (NEB) at 37°C. The restriction digestion–ligation samples were purified using the Agencourt AMPure XP System. Each clean read was checked using a Perl script to identify whether a read begins with a TAA site that can be recognized by the restriction enzyme MseI. The percent completeness of enzyme digestion equals the number of clean reads that contain a TAA site divided by the total number of clean reads times 100. The efficiency of enzymatic digestion for each sample was calculated in this manner. PCR amplifications were carried out in a single tube with purified samples and Phusion Master Mix (NEB) after adding universal primer and index primer to each sample. The PCRs were purified using Agencourt AMPure XP (Beckman) and pooled, then run out on a 2% agarose gel. A Gel Extraction Kit (Qiagen) was used to isolate 220–450 bp fragments (with indexes and adaptors). These fragments were then purified using the Agencourt AMPure XP System, and the resulting products were diluted for sequencing. Finally, paired‐end sequencing was performed on the selected tags using an Illumina 2500 platform (Illumina) by Novogene Bioinformatics Institute.

### 2.3 Genotype calling and SNP identification

Raw reads were estimated for GC percentage (%) and phred score (Q30), and then quality‐filtered using Stacks v2.55 with min_maf = 0.05 and max_obs_het = 0.70 (Catchen et al., 2013). After quality control, clean reads were mapped to the TKS reference genome sequence (dandelion line 1151) (Lin et al., 2018) using BWA 1.0 with parameters “mem ‐t 4 ‐k 32 ‐M” (Li & Richard, 2009). Then, sequence alignment SAM files were further converted into binary BAM files with SAMtools (Li et al., 2009). Picard were used to remove the potential PCR duplications for reducing the mismatches generated. The sorted BAM files were eventually used to perform SNP calling by using SAMtools and GATK4.0 softwares. A filter was performed to ensure the accuracy of SNP variants by using VCFtools with the following parameters: ‐‐maf 0.01 ‐‐max‐missing 0.7 ‐‐min‐alleles 2. Finally, only these high‐quality SNPs were taken for further analysis.

### 2.4 Population structure analysis

The filtered high‐quality SNPs (total 524,812) were used to perform the TKS population genetics analyses. The phylogenetic tree of 58 TKS individuals was reconstructed using the MEGA‐X (Kumar et al., 2018) by neighbor‐joining algorithm under the *p*‐distance model with 1,000 bootstrap. To carry out the population structure analysis, admixture software (Zhou et al., 2011) was applied based on the number of the most likelihood populations (*K* value) which was set from 2 to 10 with five iterations for each value of *K*. Both, length of burn‐in period and the number of Markov Chain Monte Carlo (MCMC) repeat after burn‐in were set at 100,000. The cross‐validation (CV) error rate of *K* value was analyzed and the *K* value corresponding to the minimum value of CV error is considered as the best‐fit *K* value based on Evanno's method (Evanno et al., 2005). PCA (principal component analysis) of total 58 individuals were performed using GCTA v1.93 (Yang et al., 2011). Stacks v2.55 was used to compute the patterns of genetic differentiation and nucleotide diversity with the weighted average of nucleotide diversity (*p*) and fixation index value (*F*_ST_) in 150‐kb windows. The genomic regions with simultaneous top 5% *p* ratios and top 5% *F*_ST_ values were selected as selective region signals. Treemix (Pickrell & Pritchard, 2012) was used to infer the patterns of TKS population splits and mixtures in the history of populations. The molecular variance (AMOVA) test was calculated using GenAlEx software (Peakall & Smouse, 2012).

### 2.5 Identification of candidate genes related to geographic differentiation

To identify potential selective signatures during TKS evolutionary process, we scanned genomic regions using fixation index value and nucleotide diversity methods. *F*_ST_ values (window size = 10 kb and step = 5 kb) and *P* value (θπ‐sample/θπ‐control) were adopted to discern the candidate regions responsible for the differentiation among the TKS populations with software vcftools v4.2 (Danecek et al., 2011) in this study. The candidate genome regions in the top 5% of empirical distribution of *p* and significantly higher *F*_ST_ were considered as strong selective sweep that may associate with geographic differentiation or natural selection. Genes overlapped with these strong selective sweeps were then defined as candidate genes associated with geographic differentiation. The corresponding Tajima's *D* values of each selective genomic region were estimated by ANGSD (Korneliussen et al., 2014). The GO and KEGG pathway‐enriched analysis of candidate genes were carried out using AgriGO v2.0 (Tian et al., 2017) and KOBAS 2.0 (Xie et al., 2011), respectively.

## 3 RESULTS

### 3.1 Agronomic traits diversity and cluster analysis

**FIGURE 1:**
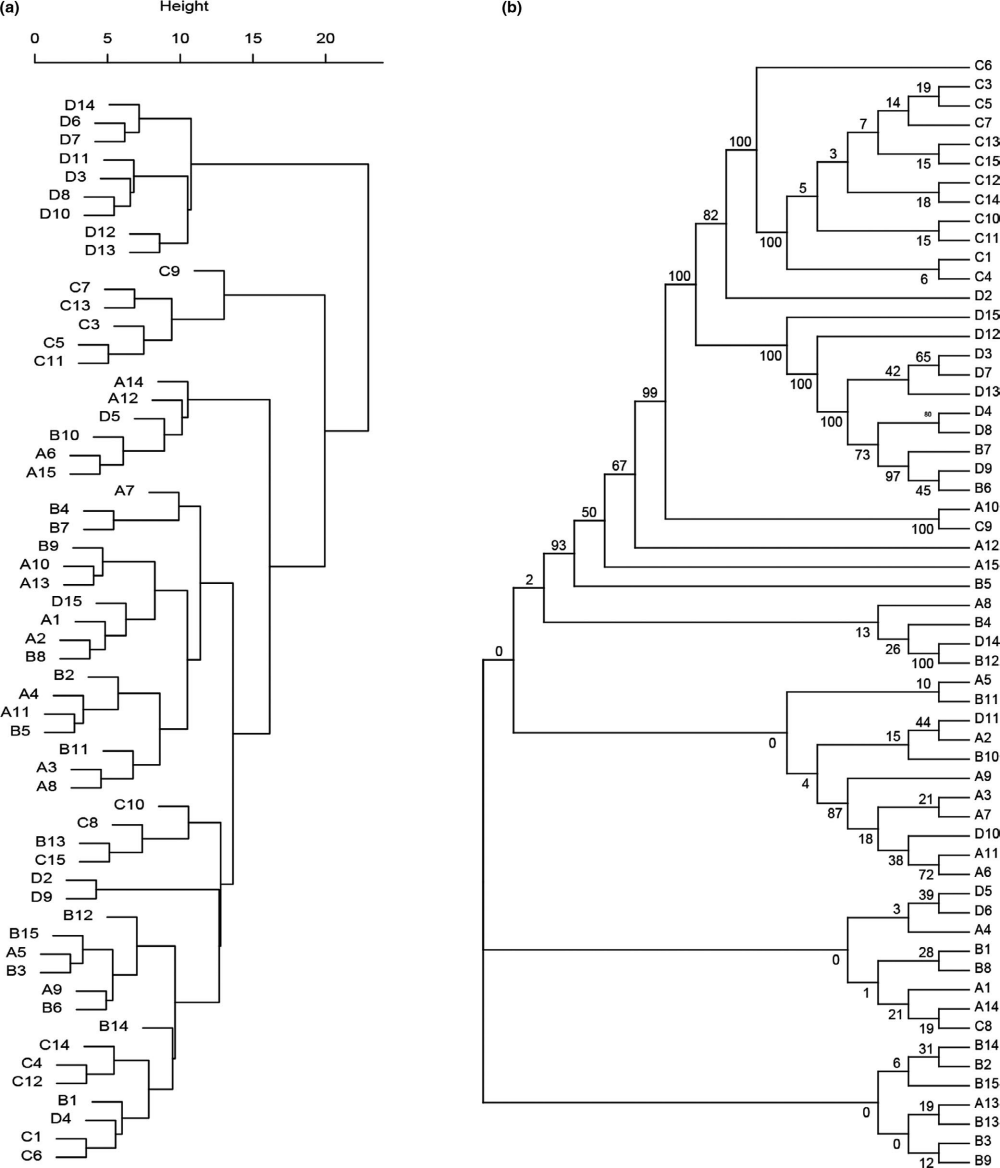
Dendrogram of cluster analysis for 58 TKS individuals. (a) Dendrogram of cluster analysis based on the morphological traits. 58 TKS individuals were collected from four different geographical regions worldwide (population A, meadow in Zhaosu County of Xinjiang; population B, Tekes River wetland in Zhaosu County of Xinjiang; population C, Tekes River wetland in Tekes County of Xinjiang; and population D, Tuzkol lake in Kazakhstan). The clustering height is the value of the criterion associated with the clustering method. (b) Neighbor‐joining (NJ) tree of 58 TKS individuals collected from four different regions. The phylogenetic tree was constructed by neighbor‐joining algorithm under the *p*‐distance model with 1,000 bootstrap using the MEGA‐X software

The ANOVA results of eight agronomic traits showed that 58 TKS individuals were significantly different in terms of their agronomic traits. The highest range of phenotypic variance (60.37) was observed in BF, whereas the least value (0.21) was attributed to LT (Table 1, Table S1). The highest phenotypic coefficient of variation (42%) was denoted to SN (Table 1, Table S1). Scape yield of per plant ranged from 3 to 30 (Table 1, Table S1). The high values of CD and LL were in TS location, and the high BF value was found in Kazakhstan. Locations TS and Kazakhstan also showed the highest LW and LT. For SN, SL, and SD, the values were evenly distributed among the individuals in different locations (Table 1). Clustering of 58 TKS individuals based on their agronomic traits revealed that they were divided into three groups (Figure 1a). First group (I) were exclusively Kazakhstan population (D) individuals, nine out of 58 individuals studied here. Second group (II) possessed about 10% of all individuals, only including six individuals in population C. Third group (III) made 75% of the individuals, from all geographical regions, including populations A, B, C, and D (Figure 1a). Based on their values for CD, BF, LL, LW, and LT among the 58 evaluated individuals, populations C and D were assigned to separate clusters, respectively.

**TABLE 1:** Statistics analysis for agronomic traits of TKS in the study

Traits	Mean ± SD^2^	Range	Variance	Skew	Kurtosis	CV (%)
CD (cm)	18.59 ± 5.97	7.41–35.39	35.59	0.85	1.05	32
BF (day)	332.86 ± 7.77	325–355	60.37	1.54	1.35	2
LL (cm)	8.96 ± 3.18	3.66–16.54	10.14	0.58	−0.45	36
LW (cm)	2.09 ± 0.62	0.90–4.10	0.39	1.13	2.26	30
LT (cm)	1.54 ± 0.46	0.76–2.79	0.21	1.07	0.82	30
SN	13.10 ± 5.51	3–30	30.34	0.67	0.80	42
SL (cm)	14.67 ± 4.77	5.31–28.80	22.75	0.14	0.31	33
SD^1^ (cm)	2.02 ± 0.58	0.80–3.19	0.33	0.08	−0.41	29

Abbreviations: BF, beginning of flowering; CD, crown diameter; CV, coefficient of variance; LL, leaf length; LT, leaf thickness; LW, leaf width; SD^1^, scape diameter; SD^2^, standard deviation; SL, scape length; SN, scape number.

### 3.2 Genomic variants of TKS population

In this study, 58 TKS individuals from four different regions in the world, including Zhaosu County of Xinjiang in China, Tekes County of Xinjiang in China, and Kazakhstan, were selected to explore the genomic diversity by GBS sequencing (Table S1). Based on the sequencing results, a total of 5,038,162 SNPs were obtained, including 524,812 (10.42%) high‐quality SNPs (MAF >0.01). Of these 524,812 high‐quality SNPs, 64,661 (12.32%) were located in protein‐coding gene regions, 386,709 (73.69%) were located in intergenic regions, and the remaining 45,859 (8.74%) were located in upstream or downstream 1 Kb regions of the identified gene coding regions. In the protein‐coding gene regions, there were 34,781 synonymous, 29,012 nonsynonymous, 199 splicing, 45 stop‐loss, and 823 stop‐gain SNPs. SNP transitions and transversions were 269,813 and 175,412, respectively. For all SNPs, intergenic variation had the highest level (73.69%), whereas intronic variation had the lowest level (5.22%) (Table S2).

### 3.3 Population diversification in TKS germplasms

**FIGURE 2:**
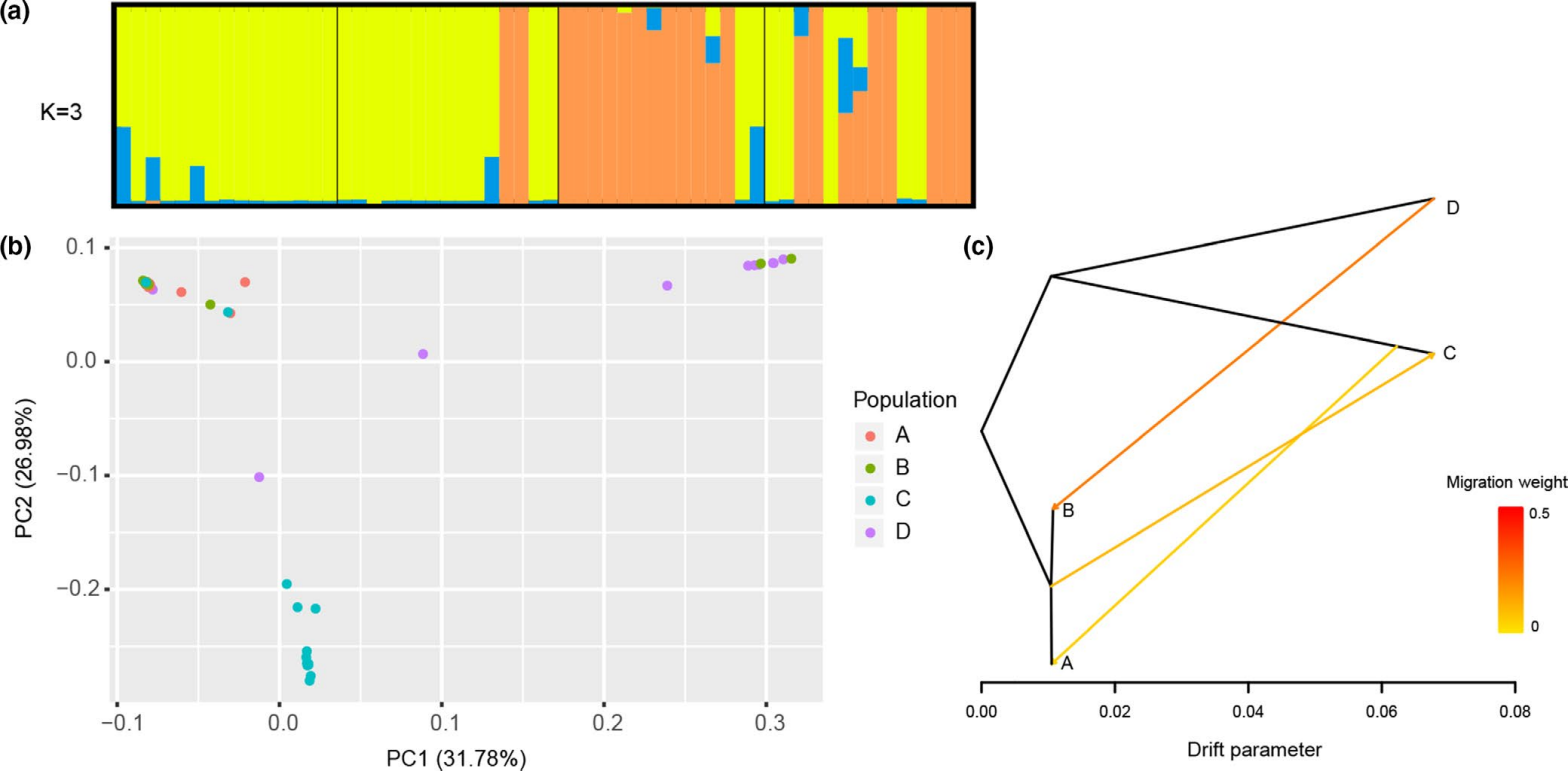
TKS population structure based on GBS. (a) Proportion of ancestry for each individual (*K* = 3). Single vertical line represents an individual accession, and different colors represent inferred genetic clusters PC1/PC2 (identify the alleles/loci). Segments of each vertical line show extent of admixture in an individual. (b) Principal component analysis (PCA). (c) Gene flow of TKS germplasm between four different regions

AMOVA analysis showed that the variation distributed within TKS populations (53.30%) and among TKS populations (46.70%) were similar (Table S4), whereas significant differentiation (*F*_ST_ > 0.15) existed between different TKS individuals. For instance, A and B populations were significantly different from C population. D population was significantly different from A and C populations, and still be significantly different with B population (Table S5). To further clarify the genetic relationships among 58 TKS individuals, high‐quality SNPs were used to investigate phylogenetic relationships. The cross‐validation error of *K* values setting from 2 to 10 indicated that the highest peak occurred at *K* = 3 and it was considered as reasonable modelling choice. Combined with the neighbor‐joining (NJ) tree (Figure 1b), population structure (Figure 2a), and principal component analysis (Figure 2b), these TKS individuals could be divided into three groups: group I contained the majority of individuals from C population (12, 85.71%); group II included the majority of individuals from D population (9, 64.29%) and two individuals in B population; and group III contained all individuals in A population and most individuals in B population (13, 86.67%), as well as a few individuals in C and D populations (Figures 1b and 2a,b). These findings are consistent with the results of population genetic diversity analysis based on Tajima's *D* value, which indicated population sizes in A and B locations expanded, whereas population sizes in C and D locations shrank suddenly. Moreover, significant gene introgression from A and B populations to C population, C population to A population and D population to B population (Figure 2c) were detected by TreeMix model. Phylogenetic analysis found that 58 wild TKS individuals have a complex genetic evolution relationship (Figure 1b). Twelve individuals in C population formed a single clade, nine individuals in D population and two individuals in B population formed a single clade, whereas the remaining individuals were clustered into another group with an admixture. In addition, population structure and PCA analysis were carried out and results evidenced a strong genetic differentiation of TKS in Kazakhstan and Xinjiang area (Figures 1b and 2a,b).

### 3.4 Candidate genes of natural selection regions during TKS evolution

**FIGURE 3:**
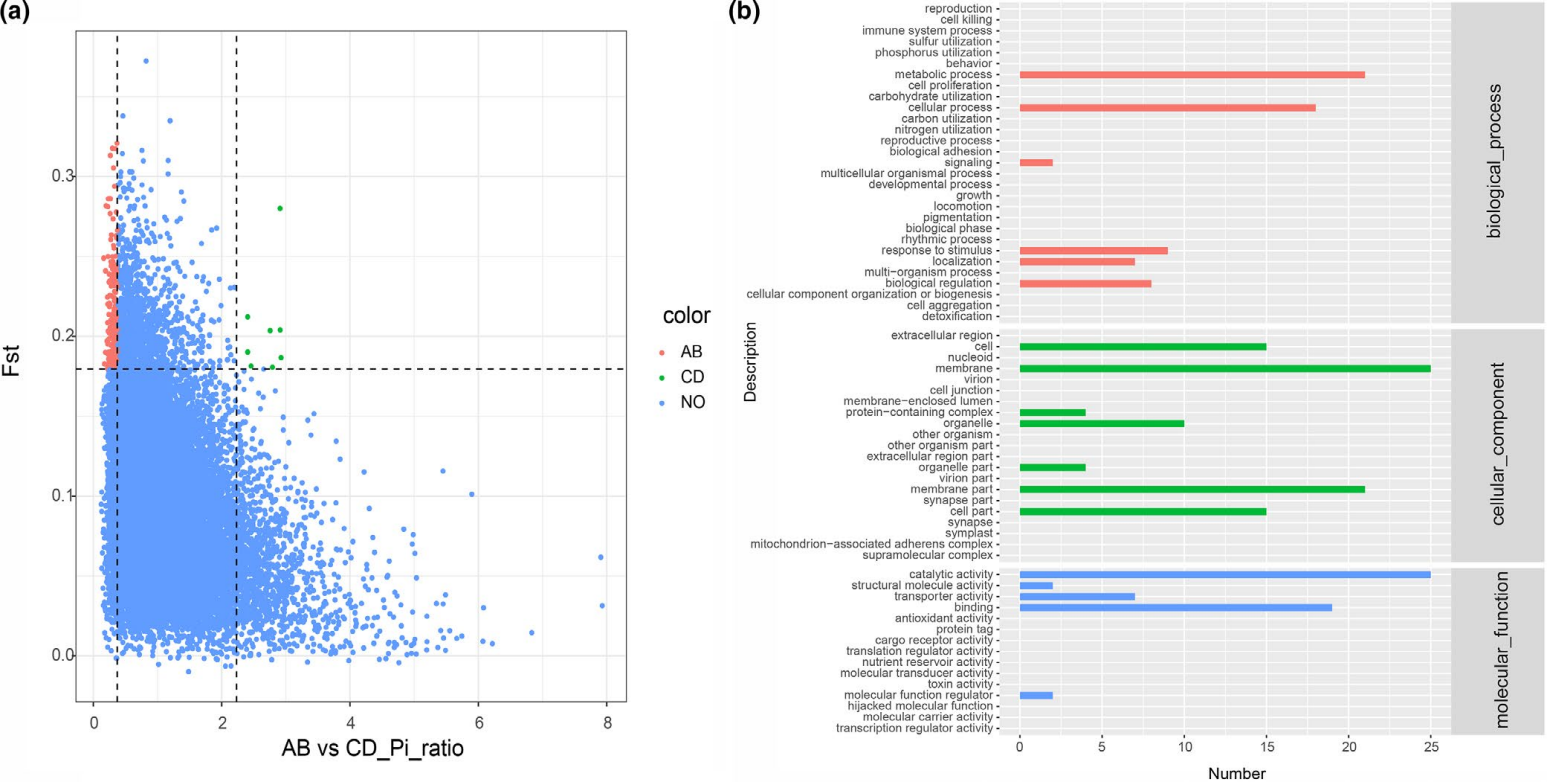
Genome‐wide screen and genes function annotation of natural selection sweeps. (a) Whole‐genome analysis of the selective sweeps through the comparison of AB population and CD populations. The genome‐wide thresholds of 2.2318 and 0.1916 were defined by the top 5% of the nucleotide diversity and *F*_ST_ values. (b) GO functional annotation of 80 genes in the identified selective sweep regions

**FIGURE 4:**
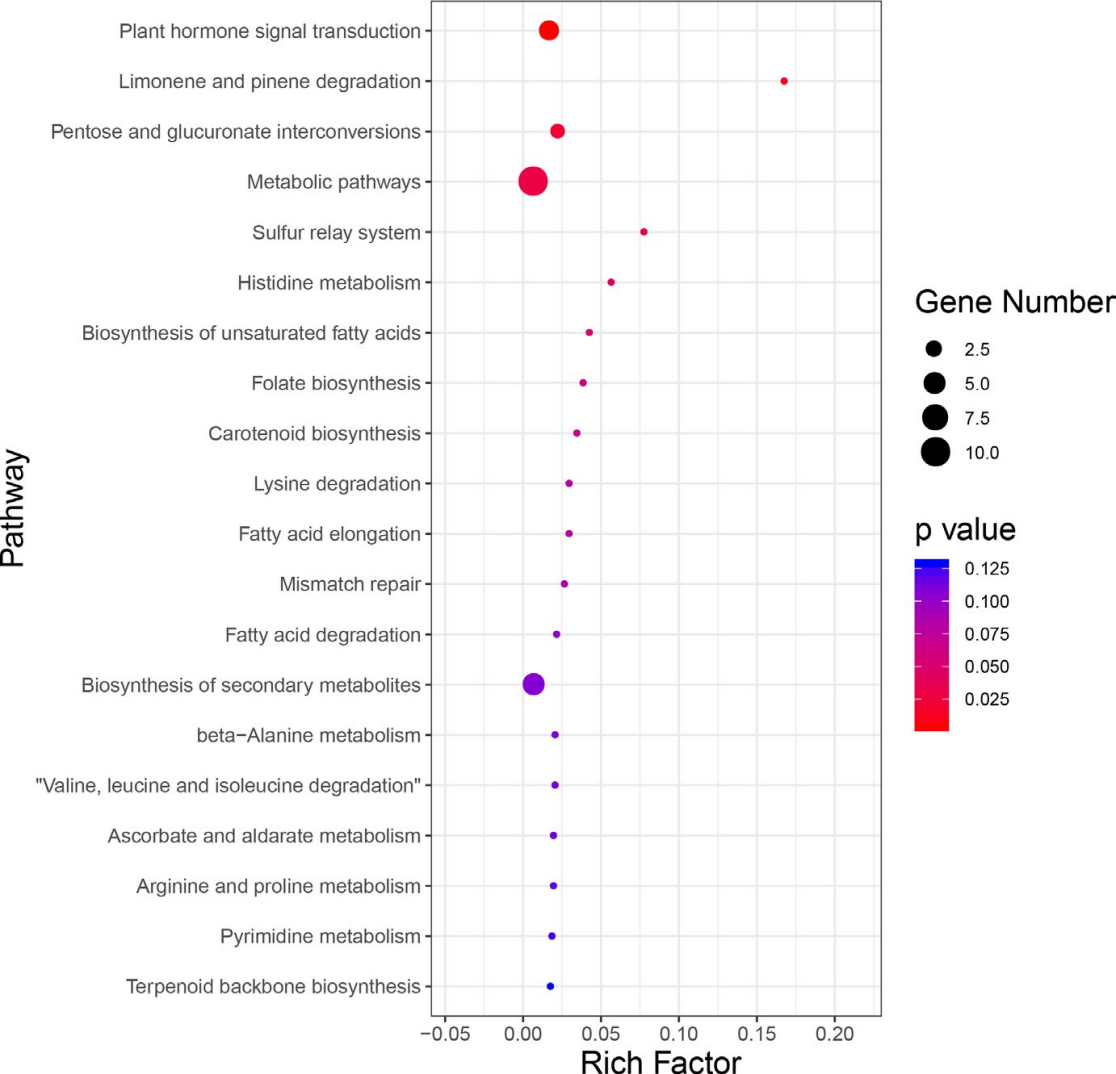
KEGG pathway enrichment analysis of 80 candidate genes in the identified selective sweep regions of AB population. Rich factor refers to the number of foreground genes concentrated in this term/the number of background genes concentrated in all genes in this term

To identify potential selective sweep during wild TKS evolutionary process in geographically distant Kazakhstan and Xinjiang, the distribution of *p* and *F*_ST_ were used and the genome regions with extremely low or high *p* ratio and high *F*_ST_ value (top 5% θπ‐sample/θπ‐control ≥ 2.2318 or θπ‐sample/θπ‐control ≤ 0.3733 and *F*_ST_ ≥ 0.1916) were considered as strong selective sweep. These results suggested that genome regions in AB population affected by natural selection have a lower level of polymorphism (median θπ‐sample/θπ‐control = 0.89) compared with CD populations. There are many genomic regions with strong selective sweep signals in AB population (0.40% of the genome and containing 80 genes) compared with CD populations (0.02% of the genome and containing 0 genes) (Figure 3a), which reflects a relatively higher inbreeding under natural selection and thus fewer recombination events and skewed allele frequency spectra in AB population compared with CD populations. To further understand the potential functions of these genes in selective sweep regions of AB population, GO and KEGG pathway enrichment analysis were implemented. GO analysis showed that most candidate genes were mainly enriched in these GO terms such as catalytic activity, transporter activity, structural molecule activity, and molecular function regulator (Figure 3b). KEGG enrichment analysis indicated that the top two enriched pathways were plant hormone signal transduction and limonene degradation (Figure 4; Table S6).

## 4 DISCUSSION

Diversity evaluation of TKS germplasm in different regions provides important breeding information for the utilization of genetic diversity. However, compared with other crops, the population genomic research of TKS has been largely limited due to lack of applicable abundant molecular markers. The combination of SNP molecular markers and agronomic traits for evaluating the genetic diversity of species is helpful to precisely analyze genetic diversity and population structure within species (Ambreen et al., 2018; Arzani & Ashraf, 2016). This method has been widely used in genetic diversity analysis in various crops such as safflower (Golkar et al., 2011), *Nigella sativa* L. (Golkara & Nourbakhshb, 2019), and *Triticum urartur* (Wang et al., 2017), whereas the genetic diversity of TKS has not been studied by this method nowadays. Therefore, 58 TKS individuals were collected in this study and divided into three different groups based on SNP molecular markers and agronomic traits. 80 candidate genes under natural selection for TKS germplasm adaptation to the Yili River Valley were further identified.

Based on the agricultural morphological data, there is a logical similarity between the TKS individuals assigned to the same group and their geographic locations, although a few individuals in C and D population were joined into population AB. It was consistent with the results of phylogenetic tree and principal component analysis based on SNPs molecular marker. And gene introgression from C population to A population and D population to B population also occurred in this study. Hence, we speculate the phenomenon could be due to the influence of various factors, such as stable genetic mutations, substitution or mixture of germplasm across the areas that migrate over long distances, inter‐regional plant material exchange, gene flow, climate adaptation and environmental influences on genetic variation (Ramanatha & Hodgkin, 2002) or the profound influence of environmental factors, similar to reports of wheat (Najaphy et al., 2012) and safflower (Golkar et al., 2011). Previous research found that common origin, convergent evolution, and subsequent natural selection may result in accessions from separate regions clustered in a common group (Reeves et al., 2012). Additionally, mutations, recombination, the number of active alleles, genetic drift, and genetic structure also could influence the amount of variation within a population (Ambreen et al., 2018). It is important to obtain a lot of individuals representing the highest possible genetic distance from the entire TKS collections (e.g., Kazakhstan genotypes and Tekes genotype in Xinjiang), which would contribute to their extraction of genes useful for breeding and exploitation of genetic resources. Not surprisingly, the highest similarity between TKS individuals in Kazakhstan and Tekes County in Xinjiang was closely dependent on the smallest genetic distance.

Population structure analysis suggested our entire TKS collection could be clustered into three groups. A few individuals from Kazakhstan and Xinjiang Tekes, as well as all individuals in population A and most in population B, were considered as one group, which is consistent with the results of other clustering methods mentioned above. The result revealed a difference in the genetic structure of TKS individuals grown in Kazakhstan and Tekes County in Xinjiang compared with the other individuals grown in Xinjiang Zhaosu County. Meanwhile, genetic admixture was also found in accessions from populations C and D to population AB, which consistent with the flow of the Tekes River from west to east. Population genetic diversity analysis indicated that population AB expanded, whereas populations C and D shrank. The frequent human activities and the destruction of the wild vegetation in there may lead to the decrease in the genetic diversity of TKS populations in Zhaosu County of Xinjiang. Recently, the ecological environment of Zhaosu County has improved (Ahan et al., 2017) and the population may have expanded after the temporary bottleneck. The admixture among individuals was also reported in other crops such as *Nigella sativa* L. (Golkara & Nourbakhshb, 2019), safflower (Ambreen et al., 2018), and *Simrouba glauca* (Kumar & Agrawal, 2017). Comparing cluster results based on agronomic traits with those based on SNPs, it shows that they were almost the same. However, the limited exposure of SNPs to artificial selection, and the coverage of SNP molecular markers to coding and noncoding genomic regions, could sometimes lead to a lack of similarity between molecular markers and morphological variations. Moreover, the top two KEGG‐enriched pathways of candidate genes in genomic regions with strong selective sweep signals in AB population were plant hormone signal transduction and limonene degradation. Previous studies have shown that there is a signal transduction network between plant hormone regulation and plant environmental adaptability, that is, plant hormone interactions can regulate plant adaptation to the environment (such as resistance to freezing, high temperature, salt, etc.) (Hu et al., 2017; Kurowska et al., 2020; Wingler et al., 2020). Therefore, the genetic diversity and selection pressures may enable the TKS to adapt to a variety of environmental conditions in different geographical regions.

## 5 CONCLUSIONS AND OUTLOOK

The results in this study revealed the potentials of SNP markers in evolutionary studies, including genotype distinctness and population genetics. Meanwhile, it points out the scope and direction of further TKS research, such as genome‐wide association study of rubber contents traits based on high‐throughput sequencing by using a wide range of accessions from each geographical location. The good varieties of TKS individuals could improve the rubber production capacity worldwide. With the increasing demand for NR and limitations of *H*. *brasiliensis* production systems, genetic engineering approaches to generate NR‐enriched genotypes of alternative NR plants based on the identification of rubber candidate genes are of great importance.

## CONFLICT OF INTEREST

None of the authors have conflicts of interest.

## AUTHOR CONTRIBUTION

**Yan Zhang:** Data curation (lead); Formal analysis (equal); Writing‐original draft (lead). **Hailong Ren:** Funding acquisition (equal); Resources (equal); Software (equal). **Xuechao Zhang:** Formal analysis (equal); Writing‐original draft (equal). **Li Wang:** Data curation (equal). **Qiang Gao:** Resources (equal). **Abudukeyoumu Abudurezike:** Visualization (equal). **Qingqing Yan:** Software (equal). **Zifeng Lu:** Data curation (equal). **Yonggang Wang:** Investigation (equal). **Qiuhai Nie:** Investigation (equal). **Lin Xu:** Funding acquisition (lead); Project administration (lead); Writing‐review & editing (equal). **Zhibin Zhang:** Formal analysis (lead); Project administration (equal); Software (equal); Writing‐review & editing (lead).

## Supporting information

Table S1

Table S2

Table S3

Table S4

Table S5

Table S6

## Data Availability

Sequencing data were stored in the NCBI Sequence Read Archive (SRA) under BioProject number PRJNA686722. Supplementary materials are available on Dryad (https://doi.org/10.5061/dryad.qnk98sfgh).
